# Albuminuria in Scarlet Fever

**Published:** 1871-01

**Authors:** 


### Miscellaneous.

### Albuminuria in Scarlet Fever.

FROM THE CLINIC OF PROF. OPPOLZER.

Affection of the kidneys is the most frequent complication of scarlet fever. It may occur either early or late in the course of the complaint; and generally leads to anasarca or dropsy, usually about the third week of the disease. Nephritis may come on very slowly or gradually, and is *sometimes* only recognizable by careful examination of the urine.

In other cases nephritis ensues with high fever, great debility, vomiting and sometimes diarrhœa. The lumbar region becomes tender; the urine scanty, cloudy, brown-red, and containing red blood-cells; its specific gravity generally high, and containing albumen in greater or less quantity. Characteristic of the complaint is the appearance of casts of the kidney tubes. But there are cases in which the first symptoms of nephritis occur *in the beginning of the complaint*, and only a few days after the eruption, though the urine is neither diminished, nor yet contains blood or albumen. Affection of the kidneys may result in ascites, anasarca, or serous effusion into the different cavities of the body, or these symptoms may be absent, and the disease show itself in the presence of convulsions, coma, and paralysis, as the consequence of uræmia.

A great difference in the character of the urine is observed *during the developement*, and during the course of scarlet fever.

*During its developement* albuminuria very seldom occurs. Yet even then a very careful examination may detect degenerated epithelial cells in considerable quantity with some blood and casts. These symptoms point to catarrhal affection of the urinary tubes, and in a great number of cases are absent. In the clinique, cases have often been observed, in which at the commencement of the disease the urine has been quite clear, and yet, on standing a short time, a soft, opalescent, mucous cloud has formed, which, under the microscope, was found to consist, for the most part, of epithelial cells, and, sooner or later, substances like casts. But *similar* appearances are found in the urine *in other acute diseases*—viz., in pneumonia, meningitis, typhus, acute peri- and endo-carditis, measles, &c.; and, with regard to them, we can only say that, in many acute febrile diseases, a metamorphosis of the tissues of the internal organs takes place, especially of the epithelial cells, which become detached and loosened. This is especially the case with the kidneys. The question is still undecided, whether these deposits in the urine are merely the result of fever, or are a specific product of the scarlet fever process. But it may be said, in cases where the scarlet fever occurs *without any feverish symptoms*, that these changes are not found in the urine, and are generally absent in slight cases of the malady.

In the case of the boy, aged 10, whose case we have lately observed, a copious urinary deposit was seen during *the first two days of the eruption*, but a microscopical examination gave no result. On the *third* day of the eruption, however, there was observed a mucous clouding of the urine, which, under the microscope, was seen to contain a few cast-like bodies, extremely transparent, and provided with fine grey granules, with isolated cloudy epithelium of the mucous membranes of the tubes. It was curious that in this case distinct traces of albumen were first found on the fifth day of the disease in the form of an opalescent layer, which continued without change, and without increasing, for eleven days. Since then the urine has been quite normal. In this case the fever was quite severe for two days, and the urinary deposits may have been the consequence of this, and not the result of a specific change.

In cases of scarlet fever in which sooner or later some complication of the kidneys takes place, sensitiveness of the lumbar region is observed; either real pain or tenderness on pressure.—*Boston Medical and Surgical Journal.*

